# Recent Advances and Prospects of Nucleic Acid Therapeutics for Anti-Cancer Therapy

**DOI:** 10.3390/molecules29194737

**Published:** 2024-10-07

**Authors:** Minhyuk Lee, Minjae Lee, Youngseo Song, Sungjee Kim, Nokyoung Park

**Affiliations:** 1 Department of Chemistry, Pohang University of Science and Technology, Pohang 37673, Republic of Korea; 2 Department of Chemistry and the Natural Science Research Institute, Myongji University, 116 Myongji-ro, Yongin-si 17058, Republic of Korea

**Keywords:** nucleic acid therapeutics (NATs), anti-cancer therapy, nucleic acid delivery, gene therapy, aptamer, RNA interference (RNAi)

## Abstract

Nucleic acid therapeutics are promising alternatives to conventional anti-cancer therapy, such as chemotherapy and radiation therapy. While conventional therapies have limitations, such as high side effects, low specificity, and drug resistance, nucleic acid therapeutics work at the gene level to eliminate the cause of the disease. Nucleic acid therapeutics treat diseases in various forms and using different mechanisms, including plasmid DNA (pDNA), small interfering RNA (siRNA), anti-microRNA (anti-miR), microRNA mimics (miRNA mimic), messenger RNA (mRNA), aptamer, catalytic nucleic acid (CNA), and CRISPR cas9 guide RNA (gRNA). In addition, nucleic acids have many advantages as nanomaterials, such as high biocompatibility, design flexibility, low immunogenicity, small size, relatively low price, and easy functionalization. Nucleic acid therapeutics can have a high therapeutic effect by being used in combination with various nucleic acid nanostructures, inorganic nanoparticles, lipid nanoparticles (LNPs), etc. to overcome low physiological stability and cell internalization efficiency. The field of nucleic acid therapeutics has advanced remarkably in recent decades, and as more and more nucleic acid therapeutics have been approved, they have already demonstrated their potential to treat diseases, including cancer. This review paper introduces the current status and recent advances in nucleic acid therapy for anti-cancer treatment and discusses the tasks and prospects ahead.

## 1. Introduction

Cancer is a disease in which genetic defects occur due to various causes, such as carcinogens, viruses, and heredity, resulting in abnormal cell proliferation. Despite decades of research, cancer still remains a great threat to human health today [1,2]. Conventional anti-cancer treatments, such as radiation therapy, surgery, and chemotherapy, have limitations, such as strong side effects due to lack of specificity and drug resistance [3,4]. In contrast, gene therapy that can correct gene defects is a promising anti-cancer therapy with a specific, continuous therapeutic effect and low cytotoxicity and immunogenicity to normal cells [4,5,6]. Even now, gene therapy strategies continue to be developed and advanced, and regulating disease-related genes by utilization of nucleic acid therapeutics (NATs) is one of the approaches to achieving effective gene therapy [7,8].

### 1.1. Overview of Nucleic Acid Therapeutics (NATs)

Nitrogen bases, which are one of the main components of nucleic acids, form hydrogen bonds between complementary bases that are called base pairs. Through interstrand base pairing, they can hybridize into double-stranded structures, and through intrastrand base pairing, they can fold to form secondary structures. This is the most basic working principle of all NATs, which will be described below. NATs are nucleic acid strands that range in length from as short as 8–50 nucleotides to as long as several thousand or more nucleotides and operate through a variety of mechanisms, including regulating gene expression or protein activity. NATs include various forms of DNA and RNA, including plasmid DNA (pDNA), small interfering RNA (siRNA), anti-microRNA (anti-miR), microRNA (miR) mimics, antisense oligonucleotide (ASO), messenger RNA (mRNA), aptamer, catalytic nucleic acid (CNA), and CRISPR cas9 guide RNA (gRNA). NATs have the potential to achieve precision medicine and long-term treatment because they target disease-related genes or proteins after being delivered intracellularly [9]. However, the application of NATs requires additional delivery strategies due to their low physiological stability owing to the presence of nucleases and low permeability to cell membranes due to a negatively charged phosphate backbone of nucleic acid [10,11]. These are part of the body’s defense mechanism against invasion of exogenous nucleic acid materials, and sometimes they may induce innate immune responses and lead to serious off-target effects. To meet these requirements, various viral vector-based and nonviral-based delivery systems, such as DNA nanostructures (DNs), inorganic nanoparticles (INPs), and lipid nanoparticles (LNPs), have been developed that can protect NATs from degradation, isolate them from the external environment, increase cell membrane permeability, and maximize delivery to target cells.

**Figure 1:**
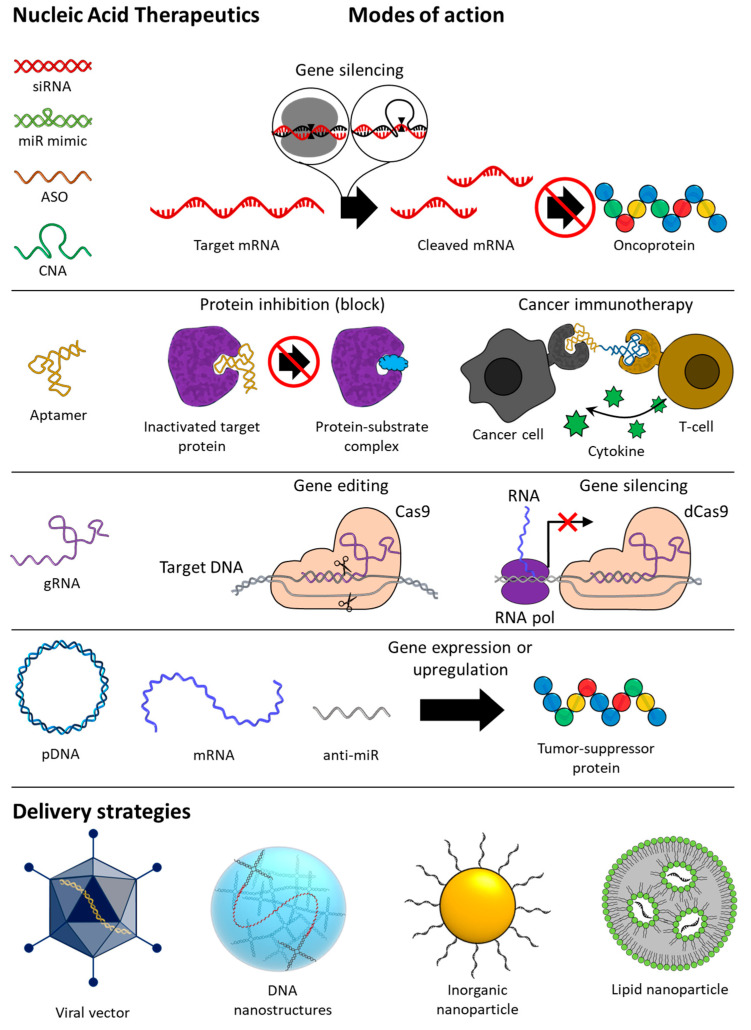
Overview of types, modes of action, and delivery strategies of nucleic acid-based therapeutics for anti-cancer therapy. siRNA, small interfering RNA; miR, microRNA; ASO, antisense oligonucleotide; CNA, catalytic nucleic acid; gRNA, guide RNA; pDNA, plasmid DNA; mRNA, messenger RNA.

In recent decades, NATs have developed remarkably along with delivery technologies, and in this review, we would like to introduce the current status and recent advances of not only NATs but also the delivery technologies for anti-cancer treatment and discuss the tasks and prospects that lie ahead. The schematic image in Figure 1 summarizes the contents to be introduced in this review.

### 1.2. Nucleic Acid Therapeutics (NATs)

Since nucleic acids play a role in storing and transmitting genetic information within living organisms, the concept of regulating the expression of genes through the involvement of exogenous nucleic acid molecules has been around since the 1970s [12]. In the 1980s, the presence of ASO in prokaryotic cells revealed that nucleic acid-mediated gene regulation is a naturally occurring mechanism within cells, which gave credence to the development of NATs [13]. Several years later, the discovery that ribozymes, which recognize and cleave RNA at specific base sequences, act as effective gene regulators provided a major advance to the study of NATs as therapeutics [14]. Since then, research on NATs has made a lot of progress, and various forms of DNA and RNA therapeutics to be described below have been developed. NATs are largely divided into four modes of action: gene silencing, protein inhibition, gene editing, and gene expression (Figure 1). Although in vivo application of NATs requires additional intracellular delivery systems, the following sections describe NATs and delivery systems separately. First, we describe the mechanism of action and target genes of each NAT, and then we describe four NAT delivery systems: viral vectors, DNs, INPs, and LNPs.

## 2. Gene Silencing

Gene silencing is a negative feedback mechanism to temporarily suppress the expression of specific genes by targeting mRNA without adding or editing genes [15]. The treatment of cancer through gene silencing aims to reduce the expression levels of genes associated with the proliferation of cancer, such as anti-apoptosis and angiogenesis. This mechanism involves the process by which NATs recognize, hybridize, and cleavage target mRNAs with complementary sequences.

### 2.1. RNA Interference (RNAi)

**Figure 2:**
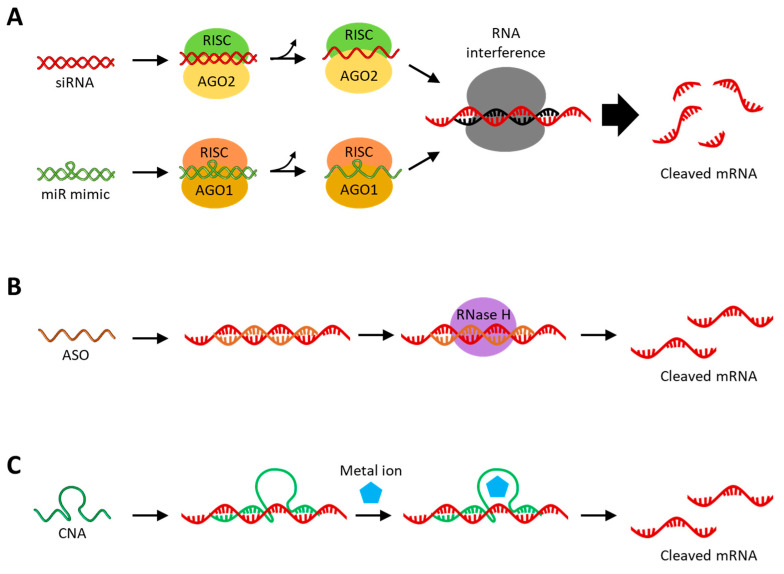
(**A**) Schematic image of gene silencing with siRNA or miR mimic. The siRNA and miR mimic are incorporated within the RISC in the cytoplasm and then unwind, activating the guide strands. (**B**) Schematic image of gene silencing with RNase H-mediated cleavage with ASO. ASOs with chemically modified sugar backbones inhibit mRNA expression through steric hindrance. (**C**) Schematic image of gene silencing with CNA-based cleavage. The presence of metal ions helps to stabilize the activated structure of ribozymes and is essential for the catalytic activity of DNAzymes.

RNAi is an intracellular negative feedback process induced by double-stranded RNA, causing the degradation of specific mRNA targets, which are defense mechanisms against external nucleic acid invasion [16,17]. siRNAs and miRNAs are non-coding RNAs that mediate RNAi and have received great attention for their high potential as therapeutics for various diseases [18]. As shown in Figure 2A, both siRNA and miRNA have RNAi activity in combination with the Argonaute protein (Ago), a component of the RNA-induced silencing complex (RISC). Although siRNA and miRNA have similar functions involved in RNAi in cytoplasm, they are of different origins. The differences between siRNA and miRNA are that siRNA is an externally derived exogenous RNA, while miRNA is an endogenous RNA derived from the genome. Additionally, siRNA inhibits a perfectly complementary mRNA, resulting in less off-target effects, while miRNA inhibits multiple mRNAs that are partially complementary to the seed sequence [7,18,19]. Naturally occurring siRNAs are derived from exogenous long dsRNAs, and long dsRNAs are cleaved in the cytoplasm by the riboendonuclease Dicer to produce siRNAs with 21 to 23 base pairs [20]. After double-stranded siRNA is loaded onto Ago2 within the RISC, one strand is selected as the guide RNA, and the other strand, the passenger RNA, is cleaved and released. In this process, the RNA strand loaded onto the Ago2 is selected based on the difference in thermodynamic stability at the ends of the two small RNA strands [21]. Synthetic siRNAs can be treated as single-stranded or double-stranded; single-stranded siRNA is unstable and thus has a relatively low RNAi effect but exhibits RNAi activity quickly, whereas double-stranded siRNA exhibits RNAi activity relatively late but exhibits a longer RNAi effect with higher efficiency [22]. Although the double-stranded siRNA has higher physiological stability, there is also a concern that off-target effects may occur, in which mRNA expression is suppressed by the passenger strand [23]. The approaches to anti-cancer therapy with synthetic siRNA can be carried out by internalizing synthetic siRNA into the target cancer cells, thereby silencing the target mRNA involved in cancer. These siRNA-target genes are mainly those that promote cell differentiation and proliferation and inhibit apoptosis, which are overexpressed in cancer cells, such as Bcl-2 [24], survivin [25], epidermal growth factor receptor (EGFR) [26], Janus kinase 1 (JAK1) [27], and polo-like kinase 1 (PLK1) [28]. In addition, the JIP1 and CLN3 genes involved in resistance to chemo drugs and the PD-L1 and CTLA-4 genes acting on immune avoidance can also be targeted by siRNA for anti-cancer therapy [29,30,31].

miRNAs are about 22 nt long RNAs that are synthesized through multiple processing steps in various pathways within living cells [32]. In the major synthesis pathway, miRNAs are first transcribed by RNA polymerase II (RNAP II) from genome to primary miRNA (pri-miRNA) with a double-stranded hairpin-loop structure, typically over 1 kb long. Afterwards, the pri-miRNA undergoes a post-transcriptional modification process, is capped and polyadenylated, and is further processed by the microprocessor complex consisting of Drosha (ribonuclease III enzyme) and DGCR8 (DiGeorge syndrome critical region 8) proteins to form precursor miRNA (pre-miRNA) having a 70–100 bp length with a 3′ end overhang [33]. The pre-miRNA is transported from the nucleus to the cytoplasm by the Exporting 5 protein, and in the cytoplasm, the 5′ and 3′ ends of the pre-miRNA hairpin are cleaved by Dicer, forming a mature miRNA duplex with about 22 bp length [33,34,35]. Among the two strands of the miRNA duplex, the one originating from the 5′ end of the pre-miRNA hairpin is called the 5p strand, and the strand originating from the 3′ end is called the 3p strand. In contrast to siRNA, miRNAs can be loaded onto the Ago protein on both strands derived from duplex, and the selection of the guide strand is influenced by its thermodynamic properties [36]. The 5p and 3p strands target different mRNAs because they have sequences complementary to each other. When one strand of miRNA duplex is loaded onto the Ago protein, the miRNA duplex is unwound to single strands, and the unloaded strand is released and degraded. Depending on the type of Ago onto which the miRNA is loaded, it either destabilizes the target mRNA or cleaves the target mRNA [37]. Anti-cancer therapy approaches with synthetic miRNAs are called miRNA (miR) mimics because they make cancer cells have the same sequence as the inhibited or inactivated tumor suppressor miRNAs. miR mimics suppress cancer cells by mimicking the biological functions of the endogenous tumor inhibitor miRNAs, such as Let-7 [38], miR-29 [39], miR-34 [40], miR-101 [41], miR-126 [42], and miR-497 [43]. Chemically synthesized miR mimics can be treated in a single-stranded form, as needed, but RNAi efficiency is reduced compared to the double-stranded form, as in the case of siRNA [44]. On the other hand, single-stranded miR mimics have relatively high cell membrane permeability compared to the double-stranded miR mimics and may be useful in reducing off-target effects [45].

**Figure 3:**
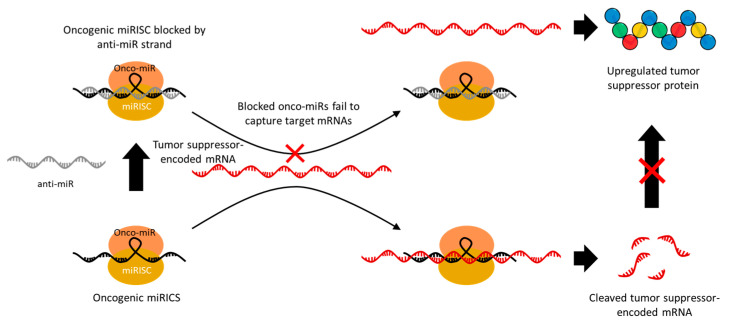
Schematic image of the mechanism of anti-miRs inducing upregulation of tumor suppressor genes by blocking oncogenic miRISC. Oncogenic miRICS inhibits expression by cleaving tumor suppressor-encoded mRNAs. Anti-miRs hybridize to oncogenic miRICS and block the gene silencing activity. As a result, tumor suppressor genes are upregulated.

Some miRNAs act as oncogenes that target mRNAs in tumor suppressor genes and are overexpressed in cancer. In this case, anti-miRNA (anti-miR) is used to irreversibly inhibit the gene-silencing activity of these onco-miRs [46]. Therefore, the use of anti-miRs leads to the upregulation of one or more tumor suppressor genes that were being inhibited by the target onco-miRs, and thus they have a significant potential for anti-cancer therapy. The anti-miR sequence is designed to have a high affinity with the miRNA; thus, it competitively hybridizes to the miRNA–RISC (miRISC) along with the target mRNA (Figure 3) [47]. The miRISCs hybridized with anti-miR can no longer cleave the target mRNA, resulting in an upregulation of the target gene expression. Among various oncogenic miRs, miR-21 in particular is upregulated in various types of cancer and has therefore been widely targeted in anti-miR-based cancer suppression studies [48,49,50,51]. miR-21 mainly causes tumor development, growth, and metastasis by downregulating the genes related to apoptosis, such as phosphatase and tensin homolog (PTEN) and programmed cell death protein 4 (PDCD4), as well as tumor growth and metastasis inhibitory genes, such as reversion-inducing cysteine-rich protein with Kazal motifs (RECK) and tropomyosin 1 (TPM1) [52,53]. In addition, miR-155 [54], miR-191 [55], miR-203 [56], miR-214 [57], and miR-221 [58] are also known to downregulate major tumor suppressor genes, which makes them a promising target for anti-miR therapeutics. Since one miRNA can effectively regulate the expression of multiple target proteins, the delivery of miR mimics related to tumor suppression or anti-miRs that block tumor-induced miRNAs has great potential for anti-cancer therapy.

### 2.2. Antisense Oligonucleotide (ASO)

ASO is a short, single-stranded deoxyribonucleotide with 12~24 nt in length that hybridizes by Watson–Crick base pairing with specific target mRNAs with complementary sequences. Upon being introduced into the cytoplasm and nucleus, ASO inhibits the translation of target mRNA, mainly through RNase H-mediated cleavage [59,60]. ASO is introduced as a single strand and hybridized to the target RNA, resulting in the formation of an RNA–DNA heterodimer, which is cleaved by RNase H (Figure 2B). RNase H is an endonuclease that is distributed in both the nucleus and cytoplasm and specifically recognizes RNA–DNA heteroduplexes and cleaves the RNA strand selectively. ASO, with a chemically modified sugar backbone, such as 2′-O-methyl (2′-OMe) or 2′-O-methoxyethyl (2′-MOE), inhibits the translation of target mRNA through steric hindrance because it interferes with the binding and cleavage of RNase H to the RNA–DNA heteroduplex [61,62,63]. These chemical modifications provide additional advantages in increasing the physiological stability of ASOs. Similar to siRNA, ASO target genes are involved in cancer differentiation, growth, proliferation, and survival, such as Bcl-2 [64], survivin [65], EGFR [66], signal transducer and activator of transcription 3 (STAT3) [67], and PD-L1 [68]. Gene silencing via ASOs has an advantage over siRNAs in that it can target precursor mRNA (pre-mRNA) or long non-coding RNA (lncRNA) within the nucleus [69,70]. lncRNAs are long non-coding RNAs with a length of greater than 200 nt involved in the regulation of gene expression through various biological mechanisms and are clearly distinguished from the highly conserved short non-coding RNAs, such as miRNAs and siRNAs [71,72]. Therefore, abnormally expressed lncRNAs are accompanied by DNA damage, immune evasion, and cellular metabolic disorders, which contribute to various diseases, including cancer [73,74,75]. Selective inhibition of abnormally expressed lncRNA via ASO is an effective approach to cancer treatment [76]. Meanwhile, pre-mRNA is one of the primary transcripts transcribed from a gene and is an mRNA that contains introns before the splicing process [77]. Sometimes, to increase the expression rate of proteins required for the disease treatment, ASOs that cause steric hindrance are hybridized to the splice site of pre-mRNA to regulate the splicing process or to the upstream open reading frames (uORFs) of mRNA [78,79,80]. In summary, this therapeutic approach uses ASO to treat cancer by suppressing oncogenic genes or increasing the expression of tumor suppressor genes by targeting various target RNAs present in the cytoplasm and nucleus.

### 2.3. Catalytic Nucleic Acid (CNA)

CNAs are short single-stranded RNA (ribozyme) or DNA (DNAzyme) molecules with biological catalytic functions, such as RNA cleavage, in combination with a metal cofactor (Figure 2C) [81,82]. A CNA-based gene-silencing strategy has the advantage of not interfering with the endogenous RNAi pathway even when treated at high concentrations because it does not rely on endogenous protein enzymes. In addition, CNAs lacking catalytic activity sometimes exhibit the gene-silencing activity through the antisense effect [83,84]. Before the discovery of CNAs, it was thought that all enzymatic processes within cells were protein-based. This assumption has changed since ribozyme was discovered in the early 1980s, in which the self-splicing RNA or the catalytic site of RNase P was an RNA moiety [85,86]. In contrast to ribozymes found in nature, DNAzymes, which first appeared in the 1990s, are selected using in vitro selection and chemically synthesized [87]. The in vitro selection process prepares a DNA library containing 10^13–^10^16^ random sequences and incubates them with the target under the applicable conditions. In the next step, nonspecifically reacting sequences are removed, and sequences that react specifically are recovered and amplified. This process is repeated for 5–15 rounds to enrich the winner DNA strands and perform sequence analysis. Finally, the sensitivity and selectivity between the winner DNA strands are compared to select the desired DNAzyme. Ribozymes can be found not only in nature but also synthesized through in vitro selection [88]. In addition to CNAs that catalyze RNA cleavage reactions, there are CNAs with various other functions, such as alkylation, ligation, and oxidation, but this review will focus on the RNA-cleaving CNAs used in gene silencing [88,89].

**Figure 4:**
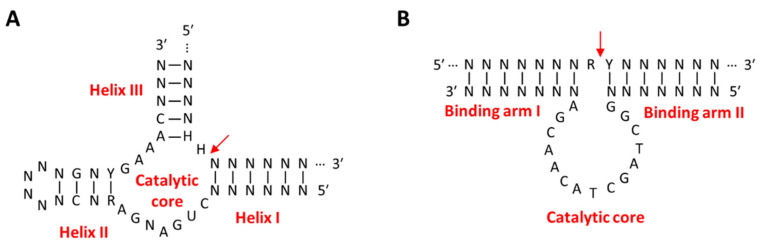
Schematic images of the secondary structure of (**A**) the hammerhead ribozyme–substrate complex and (**B**) the 10–23 DNAzyme–substrate complex. The red arrow indicates the cleavage site of the substrate RNA. (**A**) The hammerhead ribozyme consists of an intrastrand helix (helix II) and two interstrand helixes (helix I and III) generated after hybridizing with substrate RNA and a conserved unpaired catalytic core. (**B**) The 10–23 DNAzyme consists of two variable binding arms, I and II, on both sides of the conserved (15 nt) and unpaired catalytic core. R represents A or G; Y represents U (or T) or C; H represents A, C, or U; N represents A, U (or T), C, or G.

Among ribozymes, the hammerhead ribozyme (HHR) is the most extensively studied in gene therapy. The activated structure of HHR consists of a conserved catalytic core and three helixes surrounding it, out of which two helixes, helix I and III, are formed by hybridization with the target RNA (Figure 4A) [90]. The advantages of HHR as a gene-silencing agent are that the sequences of helix I and III can be easily controlled without inhibiting the activity of the ribozyme, and the total length of the ribozyme is 35 nucleotides, making it easy to chemically synthesize [91]. HHR recognizes and binds to a target RNA with a sequence complementary to the binding site and cleaves specific sites of target RNA through phosphate diester isomerization. Divalent metal cations are not essential for the catalytic reaction of hammerhead ribozyme but have been reported to stabilize the activated structure [91]. Similar to other gene-silencing agents, targets of ribozyme for anti-cancer therapy are genes that are cancer-inducing, such as mutant K-ras [92] and fibroblast growth factor-2 (FGF-2) [93], or important for cancer survival, such as γ-Glutamylcysteine synthetase (γ-GCS) [94] and multiple drug resistance 1 (MDR-1) [95]. Sometimes, ribozymes recognize cancer biomarkers, such as mutant p53 protein or human telomerase reverse transcriptase (hTERT) mRNA, and then activate cancer-killing genes, such as diphtheria toxin (DT) or herpes simplex virus thymidine kinase (HSVtk), that are delivered together to kill cancer cells directly [96,97].

RNA-cleaving DNAzymes (RCDs) are suitable for use as gene-silencing agents because they are physiologically stable compared to ribozymes, can cleave target mRNA more precisely, and are cost-effective to synthesize compared to other nucleic acid therapeutics [98]. Similar to ribozyme, RCDs have binding arms that can bind to substrate RNA on both sides of the conserved catalytic core (Figure 4B). RCDs cleave the substrate RNA bound to the binding arm through a transesterification reaction with the assistance of a divalent metal cation bound as a cofactor to the catalytic core [99]. The 10–23 RCD is suitable for intracellular gene silencing because it can be designed to cleave almost all target mRNAs with purine–pyrimidine (R-Y) junctions utilizing Mg^2+^ ions as cofactors with little effect on catalytic function [100,101,102]. Anti-cancer strategies using RCDs work by targeting genes involved in cancer differentiation, growth, and proliferation, such as Bcl-2 [103], survivin [104], early growth response protein 1 (EGR1) [105], and urokinase plasminogen activator surface receptor (uPAR) [106], or genes involved in cancer survival, such as PD-L1 [107], c-jun [108], and insulin-like growth factor II promoter 3 (IGF-IIP3) [109]. RCDs have a unique advantage over other gene-silencing agents in that their gene-silencing efficacy can be improved by adjusting the length of the binding arm, chemical modification, or using a combination of multiple RCDs [110,111,112,113]. In addition, RCDs are able to activate the catalytic core by oncogenic mRNA to simultaneously achieve down-regulation of the target mRNA, additional anti-cancer strategies, and diagnosis of cancer [114,115]. However, since CNAs are less efficient in gene silencing compared to other endogenous gene-silencing pathways, they have recently been mainly used as a trigger for drug release or as a signal generation tool for disease diagnosis.

To summarize, anti-cancer therapies through gene silencing introduced above commonly go through the following steps: (1) identifying genes involved in cancer development, (2) designing and synthesizing NATs that specifically inhibit cancer-related genes, and (3) safely delivering the synthesized NATs to the cytoplasm of target cells. Target genes include genes related to cancer growth, differentiation and metastasis, angiogenesis, immune avoidance, and drug resistance.

## 3. Aptamer

Aptamers are single-stranded RNA or DNA oligonucleotide molecules, 20 to 100 nucleotides long, that fold into specific three-dimensional structures to specifically recognize and bind specific targets. In addition to their specific three-dimensional folding structure complementarity, aptamers also provide high selectivity and binding affinity with specific targets through noncovalent interactions, such as hydrogen bonding, van der Waals forces, electrostatic interactions, hydrophobic effects, and π-π stacking [116,117]. Aptamers, also known as chemical antibodies, are similar to conventional antibodies consisting of proteins in that they not only bind to their targets but can also modulate the biological activities of the targets [118]. This suggests that aptamers can act as bioregulators and be utilized as therapeutic agents for anti-cancer therapy. In addition, aptamers have advantages over conventional antibodies, such as high thermal and physiological stability, low immunogenicity, small size, low production cost, and easy functionalization [119]. In particular, aptamer can be integrated with therapeutic factors, such as small-molecule chemo drugs, peptide molecules, and other NATs, as molecular recognition factors and can be utilized for targeted cancer treatment [120].

The first RNA aptamer was reported by Ellington et al. in 1990 and was successfully screened through the SELEX process [121]. In 1992, the first DNA aptamer was reported by Bock et al., and since then, many aptamers have been screened through SELEX [122,123]. SELEX to obtain aptamers is similar to the SELEX process of CNA. Combinatorial DNA/RNA libraries containing 1015–1016 different random sequence regions (20–60 nt) are incubated with the target followed by isolating and amplifying sequences that bind to the target, and repeating this process in 8–12 rounds to ultimately obtain sequences with high affinity for the target. As various SELEX technologies have been developed, a variety of aptamers have been screened for various targets, such as small molecules, peptides, and proteins [116]. In particular, significant progress has been made in the field of nucleic acid-based anti-cancer therapeutics by selecting aptamers that target cancer cells via cell-based SELEX [124]. In the case of aptamers, there was no difference in effectiveness between RNA aptamers and DNA aptamers, and DNA aptamers are known to be advantageous in terms of synthesis process and physiological stability [125].

Aptamer-based anti-cancer therapeutic strategies can be broadly divided into three categories: (1) interfering with the biological function of target proteins, (2) enhancing cancer immunotherapy, and (3) integrated with therapeutic agents for targeted therapy.

### 3.1. Aptamer-Based Protein Inhibition

**Figure 5:**
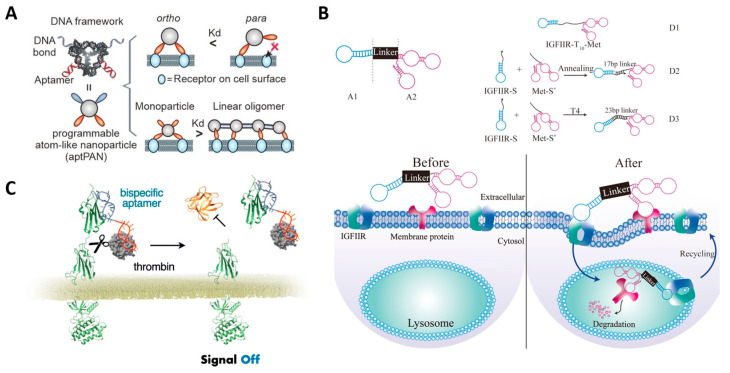
Anti-cancer therapeutic strategies using aptamer-based protein inhibition. (**A**) Schematic image of multimeric aptamer for EpCAM and PDGF protein inhibition. (**B**) Schematic image of bispecific aptamer for lysosomal degradation of target membrane proteins with IGFIIR-mediated lysosomal proteolysis systems. (**C**) Schematic image of bispecific aptamer for target membrane receptor inhibition by thrombin-mediated cleavage. (Adapted from Refs. [134,138,139]).

The anti-cancer strategy of interfering with the biological function of a target protein involves inhibiting the growth, metastasis, and invasion of cancer by binding to growth factor receptors or chemokine receptors to block cell signaling [126]. Platelet-derived growth factor (PDGF) is one of the growth factors that regulates cell growth and division. Since it especially regulates angiogenesis, which is essential for cancer growth, the overexpression of PDGF is found in various cancers. Therefore, PDGF was considered a promising target for cancer treatment, and in fact, blockade of the interaction between PDGF and PDGF receptor through aptamer resulted in the inhibition of colon cancer proliferation [127,128]. CXC motif chemokine 12 (CXCL12) is a chemokine protein, also known as stromal cell-derived factor 1 (SDF-1), that provides cancer drug resistance and immune checkpoint inhibitor resistance. NOX-A12 is an aptamer for CXCL12, which binds to CXCL12 and interferes with its interaction with CXCR4 and CXCR7 receptors [129]. When NOX-A12 was used in combination with radiotherapy, immune checkpoint inhibitors, or cytotoxic drugs, effective inhibition of cancer growth was observed [129,130,131]. The overexpression of the human epidermal growth factor receptor (HER2) is also associated with cancer growth and survival, and tumor suppression can be achieved through protein interference with aptamers targeting HER2 [132,133]. The protein inhibition efficiency of these aptamers eventually depends on the affinity between the aptamers and target proteins; hence, multiplexing of aptamers has been shown to be more effective in inhibiting surface proteins, such as HER2, PDGF, and epithelial cell adhesion molecules (EpCAM) (Figure 5A) [133,134].

In addition to strategies in which aptamers bind to target proteins and interfere with their interactions, bispecific aptamers can be utilized to control cell signaling by regulating the spatial arrangement of cell surface receptors. This strategy allows for more precise targeting because it targets multiple types of cancer-related proteins simultaneously, thereby minimizing any off-target effects [135]. The mesenchymal epithelial transition (Met) receptor is considered one of the targets for preventing cancer metastasis because it is known to be involved in cell growth and specifically in the metastasis of cancer cells. Studies have reported that pairing the Met receptor aptamer with a cancer cell biomarker, CD71 or Transferrin receptor (TfR), using a bispecific aptamer interferes with the interaction between the Met receptor and hepatocyte growth factor (HGF), thereby inhibiting cancer cell metastasis [136,137]. Han et al. reported a platform technique for inducing lysosomal degradation of target proteins by utilizing bispecific aptamers that simultaneously target Met receptors and lysosome-shuttling receptors (IGF-IIRs) (Figure 5B) [138]. Hoshiyama et al. achieved the inhibition of tumor growth signaling through aptamer-mediated cleavage using a bispecific aptamer that simultaneously targets fibroblast growth factor receptor 1 (FGFR1) and thrombin, a protease that selectively cleaves Arg–Gly bonds (Figure 5C) [139].

### 3.2. Aptamer-Based Immunotherapy

Immunotherapy is a treatment for cancer that stimulates the immune system to induce immune cells to attack cancer cells, unlike conventional cancer treatments that directly attack cancer [140]. When cancer cells appear, they are recognized by lymphocytes, including natural killer (NK) cells and T cells, and these immune cells kill cancer cells by secreting cytokines, such as Interferon-γ, and cytotoxic proteins, such as perforin, which is called tumor immune surveillance [141]. Cancer cells actively resist immune cells and ultimately escape immune surveillance through an immune editing process in which cancer cells with low immunogenicity survive and grow [142]. Immunotherapy is an anti-cancer therapy that induces cancer cell death by immune cells through neutralizing the immune suppression or immune evasion mechanisms of cancer cells or enhancing the tumor recognition ability of immune cells [143]. The major aptamer-based anti-cancer therapeutic strategies can be divided into two categories: (1) aptamers that inhibit immune checkpoints of cancer cells and (2) aptamers that improve the ability of immune cells to target cancer cells.

**Figure 6:**
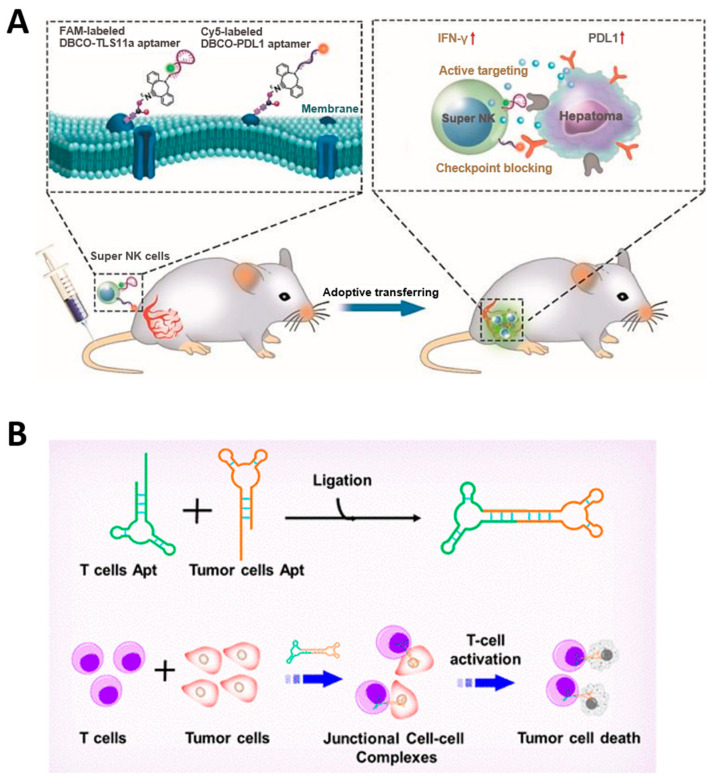
Anti-cancer therapeutic strategies using aptamer-based immunotherapy. (**A**) Schematic image of aptamer-based immunotherapy that induces apoptosis of cancer cells by blocking immune checkpoints. (**B**) Schematic image of bispecific aptamer that mediates cancer cell recognition of T cells. (Adapted from Refs. [146,149]).

The immune checkpoint is an immunosuppressive pathway that regulates the antigen recognition of immune cells to prevent the indiscriminate destruction of normal cells by excessive immune responses and is an important regulator of the immune system. PD-1/PD-L1 and CTLA-4/CD80(86) are major targets of aptamers for immune checkpoint inhibition, and immune checkpoints in cancer cells are well-known pathways for cancer to evade immune surveillance [144]. Programmed cell death 1 (PD-1) is expressed on lymphocytes, including T cells and NK cells, and inactivates lymphocytes when it interacts with PD-L1 of cancer cells. Cytotoxic T lymphocyte antigen-4 (CTLA-4) is also a T cell surface receptor that suppresses immune responses when bound to CD80 or CD86. Therefore, blocking of the PD-1/PD-L1 or CTLA-4/CD80(86) interaction via aptamer upregulates inflammatory cytokine secretion and inhibits tumor growth (Figure 6A) [145,146,147].

Immunomodulating bispecific aptamers that simultaneously target cancer cells and immune cells have the effect of recruiting immune cells around the tumor cells and accumulating them at the tumor site (Figure 6B). Fcγ receptor III (CD16) of NK cells and L-selectin (CD62L) of T cells can be targets of aptamers for recruiting immune cells, and membrane receptors overexpressed in cancer cells, such as PTK7 [148] and c-Met [149] or PD-L1 [150], an immune checkpoint, can be targets of aptamers for binding to cancer cells. Targeting PD-L1 can inhibit the immune avoidance pathway of cancer cells, further increasing the immunotherapy effect of bispecific aptamers. These immunomodulating bispecific aptamers can be categorized with [m + n] nomenclature, where [m] represents the valency of the tumor target aptamer and [n] represents the valency of the immune cell target aptamer, and higher order valencies indicate not only higher acquired affinity up to a specific upper limit but additionally acquired resistance to nucleases through steric hindrance [151].

### 3.3. Aptamer-Based Targeted Therapy

**Figure 7:**
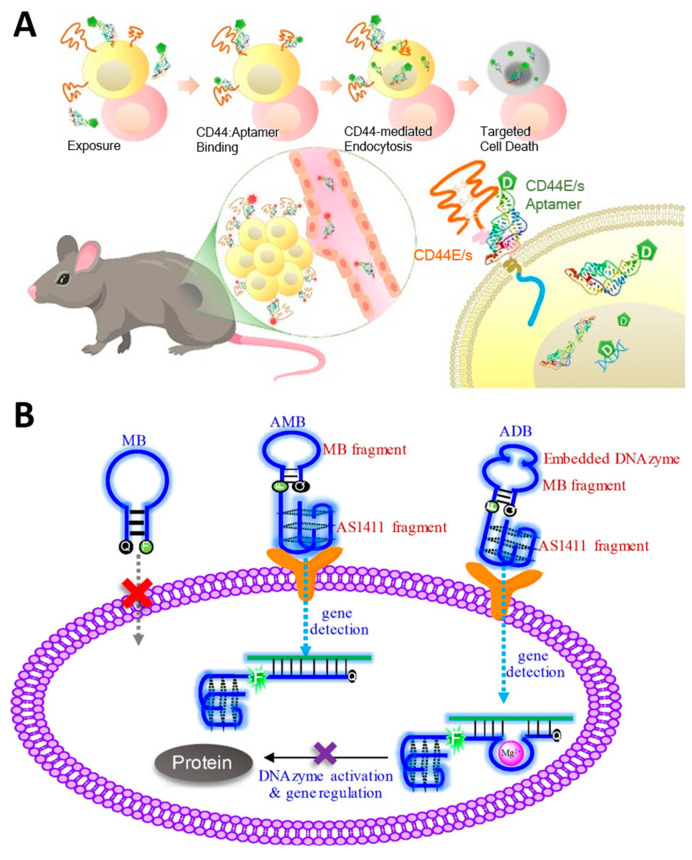
Anti-cancer therapeutic strategies using aptamer-based targeted therapy. (**A**) Schematic image of 5-Fu conjugated CD44E/s bispecific aptamer-based therapeutic for targeted delivery of anti-cancer drugs. (**B**) Schematic image of DNAzyme-embedded AS1411 aptamer that simultaneously detects and suppresses tumor genes. (Adapted from Refs. [154,163]).

The molecular recognition properties of aptamers to target specific target proteins can be combined with other therapeutic elements and used in cancer-targeting drug delivery systems. Aptamers, which can be chemically synthesized, are attractive candidates for tumor-targeting therapy as an alternative to conventional antibodies because they can be produced inexpensively and with high reproducibility, and they facilitate the functionalization of therapeutic elements at desired locations [152]. Conventional chemotherapy and radiotherapy, which have problems, such as short half-life, high side effects, immune responses, and insufficient therapeutic effects, can have a greatly improved therapeutic effect through combination with aptamer [125,153]. Lo et al. reported an aptamer-based therapeutic that effectively inhibits hepatocellular carcinoma by conjugating the anti-cancer drug 5-fluorouracil (5-FU) to an aptamer that dually targets CD44E/s, a surface glycoprotein overexpressed in cancer cells (Figure 7A) [154]. Jeong et al. developed an aptamer-based therapeutic that selectively inhibits breast cancer by conjugating maytansinoid (DM1), a selective microtubule inhibitor, to an aptamer targeting HER2 [155]. Doxorubicin (Dox) is one of the most widely used anti-cancer drugs for various solid and metastatic tumors, especially due to its intercalation properties in the nucleic acid duplex; it damages the genes in the nucleus to induce apoptosis of cancer cells, and when used in combination with NATs, it has the advantage that no additional bonding process is required [156]. Yao et al. reported an aptamer-based therapeutic that can effectively target colorectal cancer by self-assembling four AS1411 aptamers that target nucleolin overexpressed in cancer and intercalating Dox [157]. Tan et al. reported an aptamer-based breast cancer treatment that combines a chemosensitizer and an anti-cancer agent by co-delivering a peptide that specifically inhibits heat shock protein 70 (HSP70), a cytoprotective protein overexpressed in cancer, an aptamer that targets the oncoprotein mucin-1 (MUC-1), and Dox [158]. Omer et al. used trimers of A9g aptamer targeting PSMA to deliver Dox, thereby increasing the affinity for target cancer cells and resulting in higher efficacy in anti-cancer effects than when using A9g aptamer monomers [159]. Aptamer is also conjugated with radiosensitizers, such as 1,10-Phenanthroline and gold nanoclusters, increasing the radiation sensitivity of tumors and enabling radiation therapy with fewer side effects [160,161].

In addition to these chemo drugs or metal nanoclusters, aptamers can be used with other NATs, such as siRNAs, ASOs, or DNA genes. In particular, aptamers have the advantage that no additional process is required for conjugation with other NATs because they consist of nucleic acid. Dassie et al. reported significant inhibition of PSMA-positive tumors when treated with a conjugating RNA aptamer targeting prostate-specific membrane antigens (PSMA), specifically expressed in prostate cancer cells with siRNA targeting PLK1 mRNA [162]. Wang et al. conjugated the AS1411 aptamer that targets nucleolin and DNAzyme that recognizes and cleaves survivin mRNA and introduced a pair of fluorophore and quencher at both ends of the DNAzyme to enable simultaneous silencing and detection of tumor genes (Figure 7B) [163]. Strategies to attach two or more NATs to aptamers have also been attempted. Liu et al. reported a chimeric structure that combined an RNA aptamer targeting PSMA with two siRNAs targeting survivin and EGFR, demonstrating the therapeutic effect of the combined siRNA [164]. Hong et al. reported a chimeric construct in which two ASOs were introduced into the AS1411 aptamer by conjugating an ASO targeting the oncogenic gene galectin-1 and a G-rich sequence that can form AS1411 through G-quadruplex (G4) interaction [165]. In addition, anti-miR, which blocks onco-miR, is conjugated to an aptamer targeting cancer-related membrane proteins, such as EGFR and CD133 and is used as a target anti-cancer treatment [48,49].

## 4. CRISPR Cas9 Guide RNA (gRNA)

CRISPR Cas9 (Clustered Regularly Interspaced Short Palindromic Repeats associated protein 9) is an immune defense system naturally present in prokaryotes, such as bacteria and archaea [166,167]. The gRNA, also called sgRNA (single guide RNA), is a short RNA strand containing a 20 nt sequence complementary to the target DNA that complexes with the Cas protein and serves as a guide for gene editing. Unlike the first- and second-generation genome-editing tools, ZFN (Zinc Finger Nuclease) and TALEN (Transcription Activator-Like Effector Nuclease), which require protein engineering depending on the target, the third-generation tool, the CRISPR Cas system, is based on RNA-guided nucleases; hence, Cas proteins can be generally used regardless of the target, revolutionizing genome editing [168,169]. Among the CRISPR Cas systems, class II CRISPR Cas9 is most widely used in the field of gene editing because of its simplicity, efficiency, and ease of use [170].

**Figure 8:**
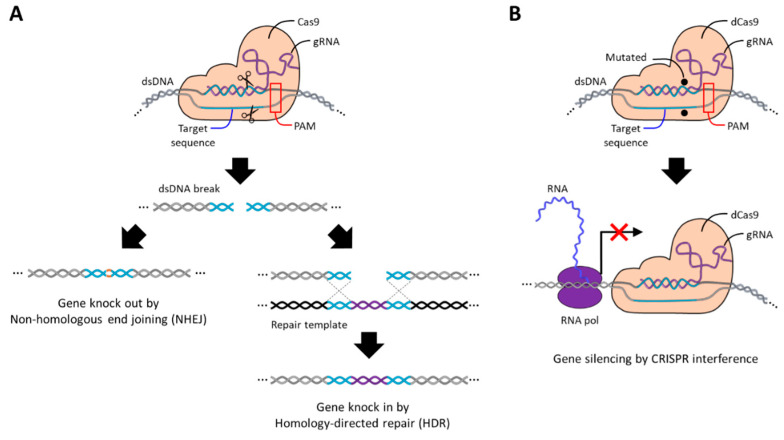
Schematic images of the CRISPR Cas9 and CRISPR dCas9 systems. (**A**) Schematic image of CRISPR Cas9 genome editing mechanism. The endonuclease domains of Cas9 (RuvC and HNH) cleave the target sequence after hybridization, and the cut DNA is repaired by NHEJ or HDR, which results in gene disruption or replacement. (**B**) Schematic image of CRISPR dCas9 gene-silencing mechanism. Mutations in the nuclease domain of dCas9 (D10A and H840A) render it unable to cleave the target sequence after hybridization and interfere with the RNA transcription process through steric hindrance.

Cas9 recognizes and cleaves target DNA, requiring a short DNA sequence 3–5 nt in length called the protospacer-adjacent motif (PAM), and compatible PAM sequences differ depending on the origin of Cas9. When gRNA hybridizes to the target DNA, the double-stranded DNA is cleaved to have blunt ends by endonuclease domains called RuvC and HNH contained in the Cas9 protein [171]. After that, the cleaved DNA site, also called double-stranded break (DSB), is repaired by a defense mechanism of cells against DNA damage called non-homologous end joining (NHEJ) or homology-directed repair (HDR) [169,172]. In the absence of additional repair templates, cells generally repair DSB through the NHEJ pathway, during which nucleotides are randomly added or removed, resulting in insertion or deletion (indel) errors that knock out target genes (Figure 8A). On the contrary, in the presence of a repair template, DSB is repaired by inserting a new gene through the HDR pathway, which is called gene knock-in. In addition to gene editing, gene silencing can be induced by suppressing the transcription of target genes through an inactive Cas9 (dCas9) lacking endonuclease activity due to the D10A and H840A mutations [173,174]. dCas9 can still hybridize with the target DNA via gRNA, but it is not able to cleave the DNA strand so it remains bound to the target gene. As a result, the transcription process of RNA polymerase for the target gene is sterically blocked by dCas9, thereby silencing the target gene (Figure 8B).

Through CRISPR Cas9, the anti-tumor effect can be greatly improved through knockout of cancer-causing genes, such as DNA methyltransferase 1 (DNMT1) gene [175], survivine gene [176], and EGFR gene [177]. Additionally, oncogenic miRNAs that inhibit tumor suppressor genes may also be promising targets of CRISPR Cas9 for anti-cancer therapy [178,179,180]. CRISPR Cas9 can also be utilized to select promising target miRNAs with anti-cancer effects [181]. Jiang et al. demonstrated that the knockout of miR-205, miR-221, miR-222, miR-30c, miR-224, miRNA-455-3p, miR-23b, and miR-505 through the CRISPR Cas9 system was effective in inhibiting the growth of prostate cancer (PCa). Genes involved in drug resistance are also promising targets for CRISPR Cas9, which exhibits effective anti-cancer activity [182]. Gene editing or gene knockout utilizing the CRISPR Cas9 system is a unique cancer treatment strategy that is difficult to achieve with other NATs. However, unlike other NATs that utilize the function of endogenous protein or the NAT itself, there are obstacles as the Cas9 protein must be delivered together with the gRNA, and it can cause fatal side effects due to irreversible DNA sequence alteration. The safety and ethical issues of CRISPR Cas9-based gene editing technology are a topic of concern for many experts, and discussions and debates surrounding them are still ongoing [183,184,185]. Despite many improvements, the gRNA of CRISPR Cas9 can form unusual hybridization with non-complementary sequences, which may lead to irreversible and lethal off-target effects. In addition, rare deletions of multiple genes have been reported during repair via the NHEJ pathway, which may lead to the development of cancer. In addition, there are ethical concerns that gene editing techniques could be applied to human germ cells to design a baby’s genetic traits.

## 5. Gene Expression

**Figure 9:**
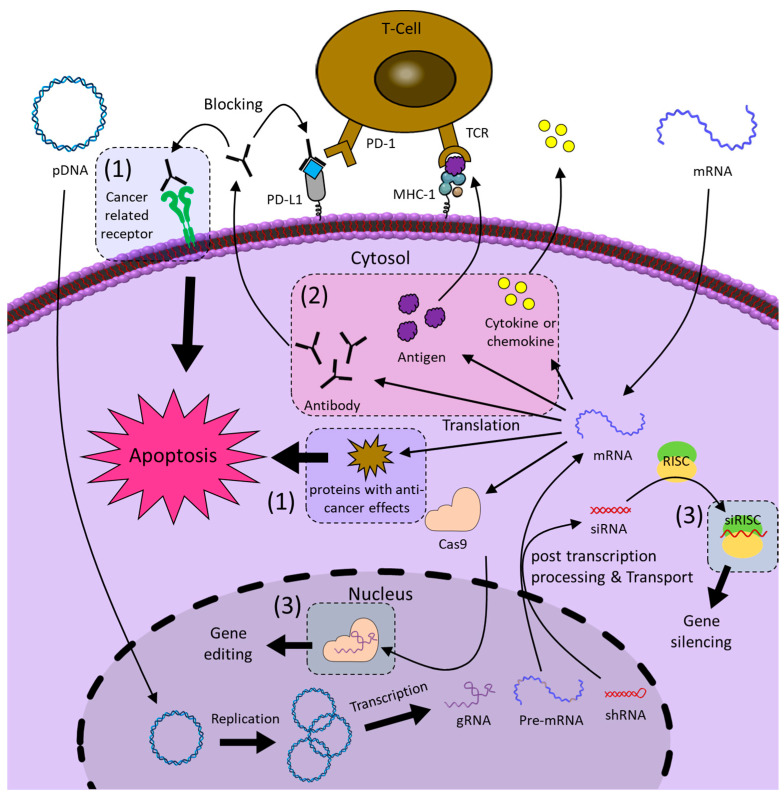
Schematic image of the anti-cancer mechanisms of pDNA and mRNA therapeutics. pDNA is delivered to the nucleus and replicated or transcribed by enzymes to synthesize RNA with therapeutic effects, such as gRNA, pre-mRNA, or shRNA, depending on the genes inserted. Pre-mRNA and shRNA are post-transcriptionally processed by enzymes into mRNA and siRNA, respectively, and transported out of the nucleus. mRNA is transported into the cytoplasm and translated by ribosomes to synthesize the encoded proteins, such as antigens, antibodies, cytokines, tumor suppressor proteins, and Cas9 proteins. The numbers represent **(1)** expression of a protein with anti-cancer effects, **(2)** DNA vaccine that induces immunotherapy by expressing an antigen or checkpoint inhibitor, and **(3)** expression of RNA-based NATs.

Gene expression is the process of creating a protein with biological functions through the flow of information from DNA to RNA and from RNA to protein. Both pDNA and mRNA are genetic vehicles that are delivered into cells to express the encoded genes. Anti-cancer therapy through gene expression of pDNA can be broadly divided into three types: (1) the expression of proteins with anti-cancer effects, (2) DNA vaccines that induce immunotherapy by expressing antigens or checkpoint inhibitors, and (3) the expression of RNA-based NATs (Figure 9). pDNA therapeutics must contain desired genetic information, and they have to reach the nucleus where RNAs can be expressed [186,187]. This transfection step is a difficult process that may reduce the therapeutic efficacy of pDNA therapeutics. On the other hand, mRNA therapeutics do not need to reach the nucleus because they can express proteins in the cytoplasm. In the case of mRNA anti-cancer therapy, it is divided into (1) tumor suppressor protein expression and (2) mRNA cancer vaccine (Figure 9). However, mRNA therapeutics have lower physiological stability than pDNA therapeutics and have a shorter duration of therapeutic effect than pDNA, which continuously produces RNA after being transfected [188].

### 5.1. Plasmid DNA (pDNA)

Plasmid DNA (pDNA) is a small circular DNA molecule found in bacteria that replicates independently of the genome and expresses genes. For therapeutic utilization of pDNA, the optimization of the plasmid backbone, including the insertion site of the desired genetic information and the arrangement of key elements, such as promoters and internal ribosome entry site (IRES), is important, which affects the immunogenicity and therapeutic effects of pDNA [187]. Transfecting the protein-encoding plasmid DNA, which has a direct anti-cancer effect, into target cancer cells can effectively suppress cancer cells (see (1) in Figure 9). Candidate proteins with such anti-cancer effects include proapoptotic proteins [189]; tumor suppressor proteins, such as p59 [190], PTEN [191], and PDCD4 [192]; cytotoxic proteins, such as saporin (SAP) [193]; proteins that enhance immunogenicity, such as RNA replicase [194]; bacterial toxins, such as diphtheria toxin [195]; and inhibitors of cancer-related receptors, such as HER2 antibody [196] and PSMA antibody [197]. Another anti-cancer strategy utilizing pDNA can induce the destruction of the cancer with the immune system by generating specific proteins called DNA cancer vaccines (see (2) in Figure 9). DNA vaccines are considered safer and more effective treatment strategies than conventional vaccines because they directly transmit the genetic information of antigens [198]. DNA vaccines use pDNA that encodes cytokines, such as interferons (INF-β, INF-γ) [199] and tumor necrosis factor-α (TNF-α) [200]; antibodies to block immune checkpoints, such as PD-L1 [201] and PD-1 [202]; and antigens, such as ovalbumin (OVA) [199], TERT [203], and glycoprotein-100 (gp-100) [204]. Next, pDNA therapeutics can be designed to express RNA-based NATs through their property to transcribe RNA after transfection (see (3) in Figure 9). short hairpin RNA (shRNA), which has an RNAi mechanism similar to siRNA, is one of the RNA-based NATs transcribed in the cell nucleus from pDNA and consists of 19–22 bp double-stranded RNA linked by short hairpins [205]. The shRNA is treated with siRNA by dicer in the cytoplasm and subsequently complexed with RISC to exhibit gene-silencing activity. pDNA encoding shRNA that silences genes involved in cancer survival and growth, such as AKT Serine/Threonine Kinase 1 (Akt1) [192], Vascular Endothelial Growth Factor (VEGF) [206], and EGFR [207], exhibits anti-cancer effects. Additionally, pDNA encoding both Cas9 protein and gRNA is a very simple and convenient strategy that avoids repetitive transfection of each component of the Cas9 system [170]. Zhao et al. achieved permanent immune checkpoint inhibition by encoding gRNA targeting the PD-L1 gene and Cas9 proteins in pDNA and observed the resultant activation of T-cell-mediated anti-tumor responses [208]. However, these strategies raise concerns that the long-lasting activity of Cas9 may increase off-target effects compared to strategies that directly deliver Cas9-gRNA complexes [209].

### 5.2. Messenger RNA (mRNA)

Messenger RNA (mRNA) is a type of RNA that carries genetic information that can direct the synthesis of proteins, and therefore it is the target of many NATs to silence genes. However, mRNA encoding for therapeutic proteins can be used as therapeutics. Unlike pDNA therapeutics, mRNA therapeutics do not need to enter the nucleus to function, making transfection relatively easy and safe [210]. In addition, mRNA therapeutics have great potential for personalized medicine because they can be produced at lower production costs in a shorter period of time than pDNA therapeutics [211]. mRNA therapeutics are considered an alternative to pDNA therapeutics, which have low transfection efficiency and can cause permanent side effects [212]. mRNA therapeutics production involves multiple unit operations, including pDNA preparation, pDNA linearization, in vitro mRNA transcription (IVT) reaction, pDNA digestion, and mRNA purification [213]. The completed mRNA therapeutic contains a 5′ cap, a 3′ poly adenine (A) tail at both ends to enhance mRNA physiological stability and protein translation efficiency, and a 5′ untranslated region (UTR), a gene of interest (GOI), and a 3′ UTR are located between them. Similar to pDNA, protein-encoded mRNAs with anti-cancer effects induce cancer cell apoptosis (see (1) in Figure 9). mRNA encoding tumor inhibitors, such as P53 [214], PTEN [215], tuberous sclerosis complex 2 (TSC2) [216], mothers against decapentaplegic homolog 4 (SMAD4) [217]; proteins that induce cell membrane disruption, such as mixed lineage kinase domain-like protein (MLKL) [218]; or cancer-related receptor inhibitors, such as HER2 antibody [219], exhibit direct anti-cancer effects. The mRNA cancer vaccine, which induces anti-tumor activity by the immune system, is currently one of the most actively studied treatments [220]. As cancer vaccines, mRNA encoding cytokines, such as Interleukin-12 (IL-12), IL-27 [221], IL-23, IL-36γ [222], and INF-α [223]; antigens, such as OX40L [219], OVA [224], cytokeratin 19 (CK19) [225], and MUC1 [226]; or antibodies that block immune checkpoints, such as anti-PD-1 and anti-PD-L1 [227], can be used (see (2) in Figure 9). In addition, mRNA encoded with the Cas9 protein is co-delivered with gRNA and used for gene editing. Wang et al. reported that the Cas9 protein-encoded mRNA is co-delivered with gRNAs that target legumain (LGMN) genes, which are known to cause various diseases, including cancer, effectively suppressing the metastatic properties of breast cancer [228].

## 6. Delivery Strategies

Although nucleic acid-based therapies have the potential to treat a wide range of diseases, including those that are difficult to treat with conventional anti-cancer therapies, their use is limited by inherent problems, such as low physiological stability and cell membrane permeability [10,11]. Moreover, exogenous nucleic acids can induce innate immune responses and cause associated off-target effects, making in vivo applications difficult [9,229]. Therefore, for effective and safe anti-cancer therapy employing NATs, an appropriate delivery system that isolates the NATs from the external environment and increases cell internalization efficiency is essential. In response to this requirement, various virus-based delivery systems and non-viral-based delivery systems, such as DNs, INPs, and LNPs, have been developed for NAT delivery. The development of a delivery system is the most important element in anti-cancer therapy employing NATs and has been extensively studied worldwide.

Among them, efforts to use viral vectors for NAT delivery have been around for 20 years [230]. Viral vectors are drug carriers that deliver NATs using a mechanism for transfecting genes into virus-infected cells. For this purpose, NATs are loaded into viruses that have reduced pathogenicity and immunogenicity. Despite recent advances in non-viral-based vectors, viral vectors still remain the most widely used delivery route for NATs, and most clinically approved NATs have used viral-based vectors [231]. Viral vectors have advantages, such as versatility for various target cells, high delivery efficiency, and large loading capacity. Viral vectors protect internal NATs with a protein shell called capsids, and some enveloped viral vectors have lipid bilayers surrounding the capsids. Viral vectors continue to improve and advance despite many potential threats and have achieved great results, with many viral vector-based therapeutics passing clinical trials around adenovirus vectors (AVs) [232]. Since the AV has a genome consisting of a long double-stranded DNA of up to 36 kb, it can insert large therapeutic genes and deliver viral DNA with high efficiency. However, since the delivered viral DNA is not integrated into the genes of the infected cell, viral gene expression is transient, and there is a risk that it may be delivered to normal cells and cause off-target effects. Furthermore, there is a disadvantage in that it exhibits strong immunogenicity [233]. In contrast, adeno-associated virus vectors (AVVs) are considered ideal vectors because they integrate into the genes of the target cells, allowing long-term expression of therapeutic genes, and have lower pathogenicity and immunogenicity than other viral vectors, including AVs. However, they have a disadvantage, as the size of the gene that can be inserted is less than 5 kb [233]. Despite continued improvements, viral vectors remain to possess relatively high immunogenicity and limited viral lifespan as they originate from viruses and are still a potential threat to the viral vector-based NAT delivery strategies [232]. These limitations make repeated administration of viral vectors difficult and lead to a decrease in the therapeutic efficacy.

Next, since DNs consist of nucleic acids, NATs can be loaded through hybridization. DNs can be considered carriers to increase the stability and intracellular transfer efficiency of NATs [234]. DNs have unique advantages as they are easy to functionalize, can precisely control the shape and size of the structure on a nanometer scale, and can carry out reversible structural transitions in response to external environments and materials, enabling controlled release. In addition, these nanostructures are composed of DNA and therefore have excellent biocompatibility and biodegradability. In particular, tetrahedral DNA nanostructures (TDNs) are widely used as drug carriers due to their strong resistance to nuclease and receptor-mediated high cell membrane permeability, thanks to their simple structure of four oligonucleotides [235]. However, since TDN is a small structure with a diameter of less than 20 nm, it has a small loading capacity and is difficult to use for the delivery of large-sized NATs, such as mRNA or pDNA. On the other hand, DNA hydrogel (Dgel) is a DNA nanostructure with a 3D network structure, and it is mostly composed of water, so it has excellent biocompatibility, high programmability, and large loading capacity, and thus has received much attention as a drug carrier [236]. Dgel has a diameter of approximately 100–200 nm and has a large loading capacity, so it has been proven that it can be used as a carrier for delivering mRNA or pDNA into cells [237,238]. Interestingly, it has been reported that compact DNA constructs, unlike loose DNA strands, have high cell membrane permeability [239,240]. In addition, DNs can easily introduce aptamers to a desired location by including aptamer sequences in the DNA strands forming the structure, and due to the flexible structural characteristics of DNA structure, additional affinity for target proteins randomly distributed on the membrane of cancer cells can be imparted through the introduction of multivalent aptamers [134,159]. DNA strands with specific sequences, such as i-motifs and aptamers, have the property of dynamically switching their structures depending on the external environment or target molecules, and this can be utilized to design DNs with controllable drug release properties [241]. It has even been reported that the i-motif sequence induces a proton sponge effect, helping DNs escape from endosomes [242]. But despite these various advantages, DNs still have a limited number of in vivo and clinical applications, and the need for further elucidation of their in vivo circulation, biodistribution, cellular endocytosis mechanisms, and endosomal escape pathways remains a challenge. Although DNs are highly biocompatible, sufficient investigation is essential to confirm whether they stimulate the immune system because living organisms react sensitively to exogeneous nucleic acid materials, and DNs are relatively resistant but are degraded by nucleases.

Next, INPs are also one of the many studied and used carriers that overcome several barriers and deliver NATs into cells [243]. INPs, such as gold nanoparticles (AuNPs), can protect surface-loaded nucleic acids from nucleases through steric hindrance and improve their intra-serum stability [244]. Additionally, it increases the cellular uptake efficiency [239]. AuNPs, in particular, are in the spotlight as nucleic acid carriers because they have advantages, such as ease of size control, surface modification, and relatively low cytotoxicity [245]. Thiol-functionalized nucleic acids are commonly used to conjugate nucleic acids to the surface of AuNPs [246]. Additionally, AuNPs can provide additional properties, such as quenching of fluorescent signals, photothermal therapy, and Raman scattering through their unique optical and electronic properties by surface plasmon resonance [247]. The SPR of AuNPs can be controlled depending on the size, shape, and assembly of AuNPs, and the SPR in the near-infrared region controlled through these ways has high biological applicability with high bio permeability. However, potential problems remain, such as the limited types of functional groups used in the conjugation of AuNPs to DNA and size-dependent cytotoxicity [245,248]. In addition, AuNPs are limited in the delivery of large-sized nucleic acid molecules, such as mRNAs or pDNAs, due to the way they load cargo onto their surfaces. Meanwhile, mesoporous silica nanoparticles (MSNPs) are also used as carriers of NATs due to their excellent properties, such as a well-defined pore size, high chemical stability and biocompatibility, and high loading capacity and surface area, as well as the ability to control the release rate through pore size control and surface modification [249,250]. Moreover, since MSNPs can adjust the size of the pores according to the size of the loaded NATs, loading large-sized NATs, such as mRNA, is also possible. Dong et al. achieved intracellular mRNA delivery by premixing cationic polymer polyethylenimine (PEI) with mRNA and then loading it onto MSNPs with a size of about 300 nm and pores of about 40 nm [251]. PEI can be used to electrostatically bind NATs to INPs, including AuNPs and MSNPs, providing the additional ability to induce endosomal escape via the proton sponge effect, but has the disadvantage of exhibiting strong cytotoxicity [252]. INPs show relatively high colloidal stability during in vivo circulation, but they are effectively removed by the liver and kidneys, and localization to the lesion remains difficult.

Next, LNPs are excellent non-viral NATs delivery vehicles with excellent NATs loading efficiency, relatively high biocompatibility, high physiological stability, high cell internalization efficiency and endosomal escape efficiency, and ease of mass production [253]. Currently, LNPs are the most studied and most advanced carriers of NATs and are helping produce a number of brilliant achievements, including combination with mRNA therapeutics to prevent the spread of COVID-19. In addition, targeted therapy with LNPs can be achieved by surface functionalization with various targeting moieties, such as antibody aptamer peptides [253]. However, the LNP is not flawless, and there is still room for development. LNPs are generally composed of helper lipids, such as cationic or ionic lipids or lipid substances, polyethylene glycol (PEG)-lipids, and cholesterol, which change immunogenicity, cytotoxicity, physiological stability, and delivery efficiency by controlling these components [254,255]. Through additional understanding of these components, the performance of LNP as a carrier can be further optimized. LNPs are spherical particles with a diameter of typically 60–150 nm, and their internal core is formed by electrostatic interactions between a negatively charged NAT and a positively charged ionic lipid at low pH; they are formed through a microfluidic system for rapid mixing. Ionic lipids are the most important components of LNPs, which have the advantage of reducing cytotoxicity and allowing long-term biocirculation by maintaining a neutral charge at physiological pH [256]. Controlling the unsaturation or length of the lipid domain of ionic lipids, or the pka value of the head group, or the introduction of biodegradable properties have been extensively studied, and accordingly, they significantly affect the properties of LNPs, such as intracellular delivery efficiency and biocompatibility. In addition, cholesterol, a helper lipid, increases the rigidity of LNP, helping with structural stability, and phospholipid, another helper lipid, helps maintain the lipid bilayer structure of LNP, helping with endosomal escape. PEG-lipid, located on the surface of LNP, prevents aggregation between particles and prevents other biomaterials from sticking, thereby playing a role in increasing colloidal stability. Additionally, adjusting or changing their ratio also greatly affects the characteristics of LNP [257]. Additionally, targeting moieties, such as antibodies or aptamers, can be conjugated to PEG-lipids or cholesterol and introduced to the surface of LNP, and multivalent aptamers can be employed to further increase the affinity for target cells due to the flexible surface properties of LNP [258,259].

## 7. Conclusions and Prospect

Since genes are made of nucleic acids, NATs can inherently affect the information flow of genes, thereby achieving anti-cancer therapy through gene expression (pDNAs and mRNAs), gene silencing (siRNAs, miR mimics, anti-miRs, ASOs, and CNAs), and gene editing (gRNAs). pDNA and mRNA have a therapeutic effect through the expression of a gene encoded with specific base sequences, while siRNA, miR mimic, anti-miR, ASO, CNA, and gRNA have a therapeutic effect through hybridization with the target nucleic acid with interstrand base pairing. Aptamers can form 3D structures through intrastrand base pairing of unique base sequences, and they can interact with specific target proteins and block their functions. Therefore, all NATs can act as therapeutics by having a specific sequence without additional chemical modification. Therefore, all NATs are made up of the same components, but by controlling the sequence, they can act as therapeutics with completely different function modes. NATs have the advantage of being easy to chemically synthesize to have specific sequences and easy to conjugate with various functional moieties, from small-molecule drugs to inorganic nanoparticles. In addition, NATs can be flexibly combined with other anti-cancer therapies, such as chemotherapy, radiation therapy, immunotherapy, and even other NATs, and have shown more improved anti-cancer effects. Therefore, NATs have unrivaled potential for long-term treatment with high efficiency for a wide range of cancers.

Despite the high potential of NATs, their application is extremely limited due to the defense mechanism of the body against external genetic material. These defense mechanisms include cell membranes that are impermeable to negatively charged nucleic acids, widely distributed nucleases from blood vessels to cytoplasm, the immune system sensitive to exogenous nucleic acid molecules, and the vascular endothelial barrier that must be passed to reach the tumor site. The conditions for an ideal NATs delivery carrier include high biocompatibility, high NATs loading efficiency, protection of the nucleic acid therapeutic from the external environment during delivery, and high cell membrane penetration and endosomal escape efficiency. To meet these requirements, various drug delivery systems have been developed and verified at the cellular level or at the in vivo level. The remarkable advancement in anti-cancer treatment strategies using NATs has been possible thanks to the advancement in drug delivery systems. The field of NATs has developed rapidly since COVID-19, and mRNA cancer vaccines are receiving particular attention. Currently, viral vectors, DNA nanostructures, inorganic nanoparticles, and LNPs are widely used as carriers of nucleic acid therapy, and among them, LNPs are the most studied as carriers for mRNA vaccines. This trend is expected to continue for the time being, and many nucleic acid-based chemotherapy drugs are expected to be approved for clinical trials in the future.

## Data Availability

No new data were created or analyzed in this study.
